# Validation of the multi-dimensional scale for Anti-Social Behavior in a higher education setting (ASB) in a sample of Afghan students

**DOI:** 10.1186/s40359-026-05383-8

**Published:** 2026-08-15

**Authors:** Mohammad Sajjad Afsharzada, Sajjad Saadat, Basir Ahmad Azizi, Wahidh Talbian

**Affiliations:** 1 Department of Education, Psychology and Social Work, Faculty of Human Sciences, Mid Sweden University, Östersund, Sweden; 2 Neuroscience Research Center, Guilan University of Medical Sciences, Rasht, Iran; 3 Department of Counseling, Herat University, Herat, Afghanistan

**Keywords:** Dari/Farsi version, Validation, Anti-social behavior, Validity, Reliability

## Abstract

**Background:**

Antisocial behavior extends across multiple layers of society, including communities, workplaces, and educational systems. Consequently, the study of Antisocial behavior in children, adolescents, and adults has attracted considerable interest from researchers across a wide range of disciplines.

**Objective:**

The present study aimed to translate and validate the Anti-Social Behavior in a Higher Education Setting (ASB) in a sample of Afghan students. The present study employed a descriptive and validation-based approach.

**Method:**

The statistical population of this study consisted of 230 undergraduate students at Herat University, Afghanistan, who were enrolled in the 2024–2025 academic year. In this study, students were selected using convenience sampling and completed an online survey (Qualtrics). To calculate test-retest reliability, 40 individuals were also selected and completed the ASB within two weeks. In this study, the ASB and the Depression Anxiety Stress Scale (DASS-21) were administered.

**Results:**

The confirmatory factor analysis (CFI) indicated that ASB had a good fit as a two-factor model (X2/df = 1.84, CFI=0.99, RMSEA=0.06). Convergent validity results showed that ASB had positive and significant relationships with depression, anxiety, and stress (*P* < 0.05). The findings also showed that ASB had good reliability, as indicated by internal consistency, test-retest reliability, composite reliability (CR) and average variance extracted (AVE).

**Conclusion:**

The findings suggest that the Dari/Farsi ASB questionnaire demonstrates acceptable validity and reliability and may be used to assess antisocial behavior among Afghan university students.

## Introduction

Antisocial behaviors are widespread across various levels of society, relationships, workplaces, and educational environments. As a result, there has been a growing interest among researchers in studying antisocial behaviors in children, adolescents, and adults [10]. Generally, antisocial behaviors emerge during childhood and adolescence and include a range of actions such as aggression and violence, theft, psychopathy, arson, vandalism of historical and artistic artifacts, and damage to public and private property [6]. Although antisocial behavior may overlap with criminal or deviant behavior, these concepts are not identical. Antisocial behavior broadly refers to actions that violate social norms, harm interpersonal relationships, or disrupt social order, whereas criminal behavior specifically involves violations of legal codes and formal laws. In university settings, antisocial behaviors may therefore include both illegal acts (e.g., fraud or hacking) and non-criminal behaviors such as academic dishonesty, harassment, hostility, or persistent rule-breaking that negatively affect the educational environment. These behaviors are also among the most common behavioral problems in children and adolescents, affecting approximately 10 to 20% of them (depending on the assessment method, sample size, and data source), and can cause serious, though sometimes temporary, issues in their social development [2]. Moreover, universities have a high potential for the emergence of antisocial behaviors, as these learning environments are socially more complex and novel compared to lower levels of education. This complexity creates more opportunities for misunderstandings and misconduct. As such, antisocial and law-breaking behaviors in educational settings are often associated with both high-risk and low-risk illegal actions, including forgery [10]. On the other hand, social media is widely used in higher education as a communication platform, creating new opportunities for interaction among students. However, the relative anonymity, rapid dissemination of content, and reduced face-to-face accountability in online environments may also facilitate antisocial behaviors such as sexting, hacking, fraud, deception, cyberbullying, and the sharing of offensive or harassing content in the form of text, images, and videos [20]. These online manifestations of antisocial behavior can negatively influence students’ psychological well-being, interpersonal relationships, and academic environments. Beyond these context-specific manifestations, previous research has identified a wide range of individual, familial, and psychosocial factors associated with the development and maintenance of antisocial behavior. Various factors contribute to the development of antisocial behaviors. For instance, adolescents exposed to mental health disorders and antisocial behavior often have a history of childhood trauma, poverty, family dysfunction, and abuse. They are also typically cared for by guardians who may struggle with issues such as substance abuse and domestic violence, and who are often long-term users of adult mental health services [12, 16]. On the other hand, mothers experiencing depression may create low-quality family interactions, inadequate parenting, and stressful home environments, all of which can contribute to the emergence of antisocial behaviors [19].

Other studies suggest that factors such as being male, possessing personality traits like risk-taking and impulsivity, hyperactivity, and feelings of loneliness can increase the likelihood of engaging in antisocial behavior [3, 4]. Furthermore, a review of previous research indicates a significant positive relationship between antisocial behaviors and psychological factors such as stress, anxiety, and depression [12, 18, 19]. In addition, dark personality traits have also been identified as influential in the antisocial behaviors of university students; higher levels of narcissism, psychopathy, and Machiavellianism are associated with an increased tendency toward antisocial behavior [10, 14].

Although numerous studies have focused on risk factors associated with antisocial behavior, other research has emphasized the importance of protective and moderating variables that may reduce the likelihood of such behaviors. Family-based self-esteem is one such moderating factor in antisocial behavior [2, 11]. An increase in empathy can also help prevent the emergence of antisocial behaviors [17]. Moreover, self-compassion and social support serve as external protective resources, while a sense of responsibility combined with a positive educational environment can reduce the likelihood of antisocial behavior [8].

In the context of Afghanistan, examining antisocial behavior among university students may be particularly important due to the rapid expansion of higher education institutions, social and economic instability, and limited availability of culturally validated psychological assessment tools. University students in such contexts may experience academic pressure, uncertainty about future employment, and social stressors that can influence behavioral adjustment and interpersonal functioning. Despite these concerns, limited empirical research has investigated antisocial behavior in Afghan higher education settings. In addition, Afghanistan’s cultural and educational context may influence both the expression and the interpretation of antisocial behavior among university students. Social norms, gender expectations, collective family values, and culturally specific understandings of interpersonal behavior may shape how students perceive actions such as hostility, academic misconduct, rule-breaking, or indirect forms of disruption. Furthermore, prolonged social instability, economic hardship, and educational challenges in Afghanistan may create unique psychosocial pressures that differ from those observed in many Western or non-conflict settings where existing antisocial behavior scales were originally developed and validated. Therefore, direct application of such instruments without cultural adaptation may not fully capture the contextual meaning and manifestation of antisocial behavior within Afghan higher education environments. Given these contextual considerations, it is important to employ assessment tools that are not only psychometrically sound but also culturally and educationally appropriate for Afghan university students.

In the history of assessing and evaluating antisocial behavior, a variety of tools have been developed and validated in countries other than Afghanistan. These include instruments such as the Illinois Bully Scale (IBS) designed for adolescents, the Achenbach Behavior Checklist for children, the Donnellan-Burt Antisocial Behavior Questionnaire, and other tools, which are typically completed through self-report or by parents or teachers. Although there are assessment tools specifically designed for children and adolescents, it is important to recognize that antisocial behavior evolves across the lifespan and varies across different environments. Therefore, there is a noticeable lack of appropriate tools for accurately assessing antisocial behavior among university students in higher education settings. Compared with instruments designed primarily for children or adolescents, the Anti-Social Behavior in Higher Education Settings (ASB) questionnaire developed by Mahmoud et al., [10] specifically focuses on behaviors relevant to university environments, including academic misconduct, interpersonal violations, and rule-breaking behaviors within higher education institutions. In addition, the scale was developed for use with student populations and reflects the social and educational realities of university settings more directly than general antisocial behavior measures. Therefore, it appears particularly suitable for adaptation and validation in the Afghan higher education context. In such environments, greater behavioral freedom and increased diversity among individuals make universities particularly susceptible to the occurrence of antisocial behaviors. For this reason, a suitable instrument to assess the quality and quantity of deviant behavior among students could serve as an effective means for preventing antisocial actions and enhancing the productivity of educational environments.

Taken together, the existing literature suggests that antisocial behavior among university students is a multifaceted phenomenon influenced by psychological, social, familial, and educational factors. Despite the growing importance of this issue in higher education settings, limited research has examined antisocial behavior among university students in Afghanistan, particularly using culturally adapted assessment instruments. Therefore, examining antisocial behavior among university students is particularly important, especially given the limited availability of culturally appropriate assessment tools for higher education settings in Afghanistan. Existing instruments were primarily developed for children, adolescents, or non-Afghan populations and may not adequately capture the contextual realities of Afghan universities. Accordingly, the present study aimed to translate and validate the Anti-Social Behavior in Higher Education Settings (ASB) questionnaire in a sample of Afghan university students.

## Method

### Methods and participants

**Fig. 1:**
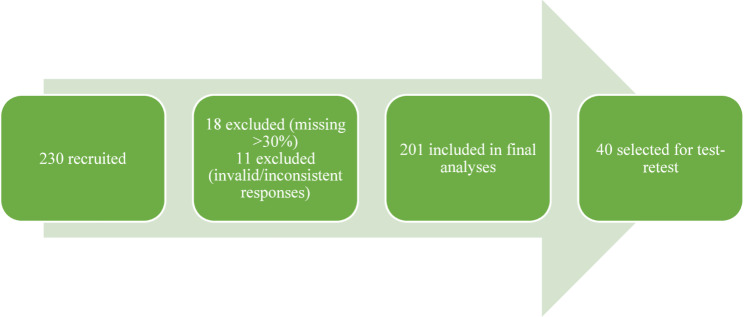
Participant flow diagram

This methodological research aimed to translate and validate the Anti-Social Behavior in Higher Education Settings (ASB) questionnaire. In this study, the construct validity was evaluated. In terms of reliability, stability, and internal consistency were assessed. The participants of this study were 201 college students from Afghanistan who were enrolled in the academic year (2024–2025). Sample size using G*Power (1 − β = 0.90, α = 0.05), the required sample size for a moderate correlation was calculated as 200 students. To prevent possible dropout, the sample size was increased to 230. A convenience sample of 230 participants answered an online survey via Qualtrics survey software (Qualtrics, Provo, UT). After data screening, 29 questionnaires were excluded from the analyses. Specifically, 18 questionnaires contained more than 30% missing responses, while 11 questionnaires demonstrated invalid or inconsistent response patterns, such as repetitive answering patterns or substantial response inconsistencies across items. Following this screening procedure, 201 valid questionnaires were retained for the final analysis (see Fig. 1). A participant flow summary describing recruitment, exclusion, and final inclusion was added to improve the transparency of the sampling process. Thus, the final sample consisted of 201 participants aged 18 to 39 years (mean age = 26.43, SD = 4.05) who were included in the study. Of these, 62.20% were female (*n* = 125) and 37.80% were male (*n* = 76). Regarding marital status, 68.20% were single (*n* = 136) and 31.80% were married (*n* = 64). In terms of employment, 66.70% were unemployed (*n* = 134) and 33.30% were employed (*n* = 67). Additionally, 40 participants, who had provided their phone numbers, were randomly selected to complete the ASB again two weeks after the initial administration to assess test-retest reliability. To evaluate test-retest reliability, 40 participants who agreed to be contacted again and provided their phone numbers were randomly selected to complete the ASB questionnaire two weeks after the initial administration. Test-retest reliability is commonly assessed using subsamples of moderate size in psychometric validation studies, and previous methodological literature has suggested that samples ranging from 30 to 50 participants may provide acceptable stability estimates [5, 7]. In the present study, the obtained test-retest coefficient exceeded the recommended threshold of 0.70, indicating satisfactory temporal stability of the ASB over the two-week interval. The inclusion criteria for the study were as follows: participants aged between 18 and 45 years, no history of psychiatric disorders, no history of divorce or parental death, and completion of an online informed consent form to participate. The exclusion criteria included incomplete questionnaires. All participants were fully informed about the study and provided their informed consent. They were also assured that their privacy and confidentiality would be maintained and that there would be no physical or psychological harm associated with their participation.

### Procedure and material

The translation of the Anti-Social Behavior (ASB) into the Dari/Farsi language was conducted using the standard forward-backward translation procedure to ensure linguistic and conceptual equivalence. The translation and cultural adaptation procedures were generally informed by established international recommendations for cross-cultural adaptation of psychological instruments (e.g [1, 13]), . Initially, two bilingual members of the research team independently translated the original English version of the ASB into Dari/Farsi. Both translators were fluent in English and Dari/Farsi and were familiar with psychological terminology and the cultural context of Afghan university students. These two forward translations were then systematically compared, and discrepancies were discussed in detail to produce a single reconciled preliminary Dari/Farsi version that best reflected the meaning of the original items while maintaining cultural appropriateness. Subsequently, the preliminary Dari/Farsi version was back-translated into English by two independent professional translators who were fluent in both languages and blind to the original ASB. The back-translated versions were carefully compared with the original English questionnaire to identify potential inconsistencies, ambiguities, or conceptual deviations. An expert panel consisting of psychologists, mental health researchers, and bilingual language specialists with experience in psychological assessment and cross-cultural research reviewed all versions of the questionnaire. Through an iterative consensus process, the panel evaluated the semantic, idiomatic, experiential, and conceptual equivalence of each item and made necessary modifications. The pre-final version of the questionnaire was informally reviewed with a small group of university students to evaluate item clarity, comprehensibility, and cultural appropriateness. Minor linguistic refinements were made based on participant feedback. This process resulted in agreement on a final Dari/Farsi version of the ASB that was deemed linguistically accurate, culturally appropriate, and suitable for use with Afghan society.

### Anti-Social Behavior in a Higher Education Setting (ASB)

The Anti-Social Behavior in a higher education setting (ASB) was developed by Mahmoud et al., [10]. The final version consists of 10 items and two subscales: Interpersonal Antagonistic Behavior (IAB) with 6 items, and Indirect Distractive Behavior (IDB) with 4 items. Scoring is based on a 9-point Likert scale ranging from “Strongly Agree = 9” to “Strongly Disagree = 1”. The two extracted factors include Interpersonal Antagonistic Behavior (items 1 to 6) and Indirect Distractive Behavior (items 7 to 10). Therefore, the total score range on this scale is from 10 to 90, with higher scores indicating greater antisocial behavior. The validity of this scale is assessed using exploratory factor analysis, which identifies two factors across the ten items. The composite reliability, Cronbach’s alpha for both subscales, is estimated to be greater than 0.70 and less than 0.90, indicating very good reliability of the questionnaire [10]. Also, in other studies, the Cronbach’s alpha coefficient for ASB has been reported as (α = 0.89) [14].

### Depression Anxiety Stress Scales (DASS-21)

The Depression Anxiety Stress Scale (DASS-21) was developed by Lovibond and Lovibond [9] to assess three related dimensions of emotional distress: depression, anxiety, and stress. The instrument comprises 21 items, evenly divided across the three subscales, with each dimension measured by seven items. Responses are recorded on a four-point Likert scale ranging from 0 to 3, where 0 indicates “Did not apply to me at all”, 1 “Applied to me to some degree, or some of the time”, 2 “Applied to me to a considerable degree, or a good part of the time”, and 3 “Applied to me very much, or most of the time”. Participants are asked to respond based on their experiences during the past week. The general score for each subscale ranges from 0 to 21, with higher scores demonstrating more serious indications. The DASS-21 is suitable for individuals aged 14 and older, including older adults, and its psychometric properties have been supported by extensive empirical research [9]. In a recent study, Neyazi et al., [15] validated the DASS-21 among a sample of 1,318 Afghan participants, reporting a high internal consistency with a Cronbach’s alpha coefficient of 0.94. The DASS-21 was used to examine convergent-related validity because antisocial behavior has been shown in previous research to be associated with emotional distress, including depression, anxiety, and stress. Although antisocial behavior is primarily a behavioral construct, affective states such as psychological distress may function as important correlates that contribute to or co-occur with such behaviors, particularly in student populations (e.g., Mehrabi et al., [12], Shakerinasab [18], . Therefore, a significant but moderate relationship was expected between antisocial behavior and psychological distress, supporting the theoretical plausibility of the ASB scale.

### Statistical analysis

Data were analyzed using SPSS-24 and AMOS-24 software. Internal consistency was assessed using Cronbach’s alpha coefficient. Test-retest reliability was assessed using the Pearson correlation coefficient. In addition, composite reliability (CR) and average variance extracted (AVE) were calculated to assess construct reliability and convergent validity, respectively. The two-factor structure of the ASB was examined using confirmatory factor analysis (CFA) to validate the construct validity of the test. Convergent validity was also calculated using Pearson’s correlation coefficient with DASS-21.

## Results

In this study, 201 college students fully responded to the online survey. In Table 1, the items and descriptive indicators of the ASB are presented. Table 1 presents the descriptive statistics for the ASB items. The skewness and kurtosis indices show that they are in the range of -2 to 2 for all items; accordingly, the data have a normal distribution. All items had a mean score close to the midpoint of the Likert scale (4), indicating that they reported moderate levels of antisocial behavior. The items also had good item-total correlations, ranging from 0.85 to 0.93, suggesting that they were all relevant to the measurement of ASB (> 0.30). Also, ASB had a positive and significant correlation with IAB (0.98) and IDB (0.97) (*p* < 0.05).

**Table 1:** Descriptive Statistics for ASB Items

Items	Subscale	Mean	SD	Skewness	Kurtosis	Item-Total Correlation
1	IAB	4.20	2.24	0.04	-1.22	0.85^**^
2		4.03	2.30	0.11	-1.34	0.88^**^
3		4.05	2.38	0.04	-1.38	0.90^**^
4		4.06	2.34	0.02	-1.34	0.93^**^
5		4.03	2.35	0.04	-1.34	0.92^**^
6		4.19	2.34	− 0.01	-1.32	0.88^**^
7	IDB	4.35	2.39	− 0.01	-1.29	0.89^**^
8		4.30	2.54	− 0.06	-1.39	0.93^**^
9		4.72	2.51	− 0.01	-1.20	0.88^**^
10		4.85	2.80	− 0.10	-1.33	0.88^**^
IAB		24.57	12.77	− 0.01	-1.40	0.98^**^
IDB		18.22	9.51	− 0.14	-1.36	0.97^**^
ASB		42.80	21.83	− 0.06	-1.40	-

**. Correlation is significant at the 0.01 level (2-tailed)

### Validity

**Fig. 2:**
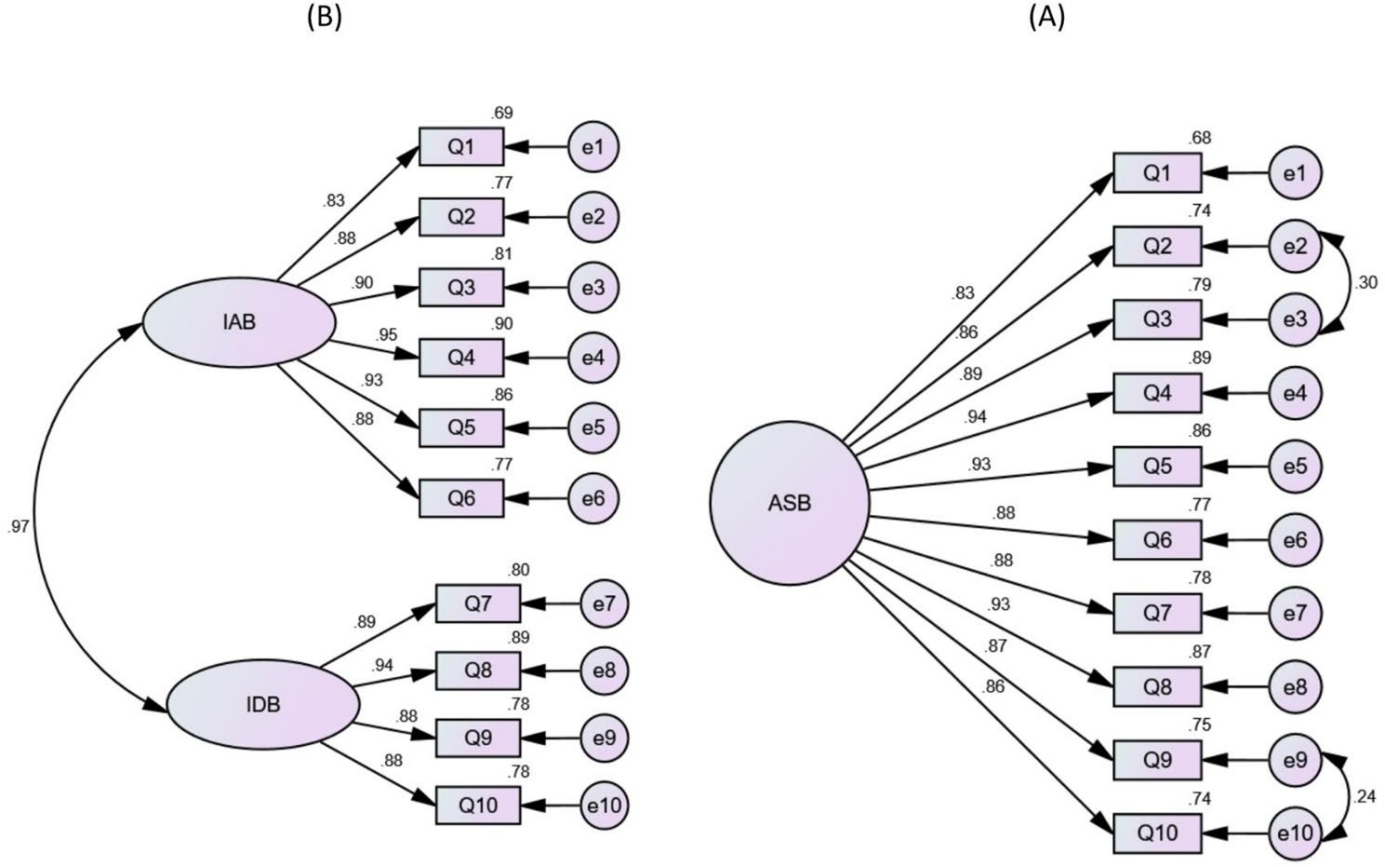
ASB measurement model as a single factor (**A**) and two factors (**B**).

Figure 2 reports the measurement model and standard coefficients.

The results of the confirmatory factor analysis in Fig. 2 in both measurement models indicate that the factor loading of all questions is greater than 0.30; the single-factor model with two modifications and the two-factor model without any modification have a good fit; the results of the fit indices are presented in Table 2.

**Table 2:** Fit indices of the CFA model in 2-factor mode

Index	Research model (1-factor)	Research model (2-factor)	Decision convergent
(X^2^)	65	62.58	*P* > 0.05
(Df)	33	34	--
(X^2^/df)	1.97	1.84	CMIN/DF < 3
Comparative Fit Index (CFI)	0.99	0.99	CFI > 0.90
Normed Fit Index (NFI)	0.96	0.98	NFI > 0.90
Tucker Lewis index (TLI)	0.98	0.98	TLI > 0.90
root mean square error of approximation (RMSEA)	0.07	0.06	RMSEA < 0.08

The fit indices showed that the two-factor model without any modification showed a good fit. The fit indices of the two-factor model are shown in Table 3, which shows that the chi-squared value (ꭕ2) is significant (*P* = 0.01) and is equal to 62.58. However, when the sample size is large, ꭕ2 is not a good indicator of model fit. In this case, the ꭕ2/df index is more valid, which is calculated as 1.84 in this research, and since it is less than 3, the fit of the model is favorable. CFI was calculated as 0.99, which indicates a good fit for the model. In this model, the RMSEA index is equal to 0.06; considering that it is less than 0.08, it indicates the optimal fit of the model. The fit indices of the single-factor model are also within the appropriate range with the modification. In fact, the single-factor model, using the proposed modification indices by creating two covariates between (item 2 with item 3) and (item 9 with item 10), provided an acceptable fit; however, considering that modification indices were required for fitting, the two-factor model is more acceptable, and the single-factor model is the next choice. Table 3 reports the convergent validity results.

**Table 3:** Convergent validity results

Variables	Depression	Anxiety	Stress
IAB	0.59**	0.66**	0.60**
IDB	0.59**	0.45**	0.61**
ASB	0.61**	0.67**	0.61**

**. Correlation is significant at the 0.05 level

The results of the convergent validity showed that ASB had a positive and significant correlation with depression (0.61), anxiety (0.67), and stress (0.61) (*P* < 0.05). Also, the IAB and IDB subscales had a positive and significant correlation with the DASS-21 subscales (*P* < 0.05). According to these findings, ASB has convergent validity.

### Reliability

Internal consistency reliability was calculated by calculating Cronbach’s alpha coefficient for the IAB subscales (α = 0.93) and IDB (α = 0.95); it was also calculated for the ASB (α = 0.97). Also, test-retest reliability was calculated with an interval of two weeks for the IAB subscales (*r*=0.74) and IDB (*r*=0.71); it was also calculated for the ASB (*r*=0.72). To evaluate construct reliability and convergent validity, composite reliability (CR) and average variance extracted (AVE) were calculated for each construct. The results demonstrated excellent reliability, with CR values of 0.96 for the IAB subscales, 0.94 for the IDB, and 0.97 for the ASB. Convergent validity was also supported, as the AVE values were 0.81 for the IAB subscales, 0.81 for the IDB, and 0.79 for the ASB. All CR values exceeded the recommended criterion of 0.70, and all AVE values were above the recommended threshold of 0.50, indicating adequate construct reliability and convergent validity.

## Discussion

This study aimed to translate and validate the Anti-Social Behavior in a Higher Education Setting (ASB) questionnaire in a sample of Afghan students. The findings from the factor analysis revealed that all items of the ASB questionnaire loaded significantly onto their respective factors, aligning with the results reported in the original scale development study [10]. In this study, two models were examined: a single-factor model (A) and a two-factor model (B). The confirmatory factor analysis of the derived models supported the construct validity of the Dari/Farsi ASB among Afghan students. These findings are consistent with previous studies [10, 14]. The confirmatory factor analysis of the obtained models indicated that the two-factor model (B) demonstrated a good fit for the data. These results highlight the questionnaire’s robust factor structure and confirm that the proposed conceptual model effectively explains the relationships among variables in a meaningful and reliable manner. From a conceptual perspective, the two-factor structure proposed by Mahmoud et al., [10] distinguishes between interpersonal antagonistic behavior and indirect distractive behavior. Interpersonal antagonistic behavior refers to actions that directly target or negatively affect other individuals, such as hostility, confrontation, or interpersonal misconduct. In contrast, indirect distractive behavior reflects behaviors that disrupt academic or institutional functioning in a less direct manner, including negligence, disengagement, or behaviors that interfere with the educational environment without necessarily involving direct interpersonal conflict. Although both dimensions represent manifestations of antisocial behavior within higher education settings, they may differ in their immediate targets, behavioral expression, and potential consequences. Therefore, the two-factor solution is theoretically meaningful and consistent with the conceptual framework proposed by the original scale developers. At the same time, the extremely high correlations observed between the two ASB dimensions suggest substantial overlap between the constructs. This overlap raises the possibility that antisocial behavior in higher education settings may also be represented by a broader general factor. For this reason, the present study examined both a unidimensional and a two-factor model. The findings indicated that both models demonstrated acceptable fit indices, although the two-factor model showed slightly superior fit and was retained for consistency with the original theoretical framework. Consequently, future researchers may evaluate whether the unidimensional or two-factor representation is more appropriate for their specific research objectives and analytical frameworks, particularly in structural equation modeling studies. Nevertheless, the very high association between the subscales warrants caution when interpreting them as fully independent dimensions. Therefore, researchers who use the two ASB subscale scores as separate variables should interpret the results with caution, as substantial shared variance may reduce the distinctiveness of the two dimensions. In studies in which differentiation among the subscales is not theoretically essential, the total ASB score may provide a more parsimonious representation of antisocial behavior. Moreover, the extremely high correlations between the ASB subscales, together with the elevated item-total correlations and internal consistency coefficients, may indicate substantial conceptual overlap among items and limited discriminant validity between the latent dimensions. Although the present study demonstrated adequate construct reliability and convergent validity through the calculation of Composite Reliability (CR) and Average Variance Extracted (AVE), future psychometric research should further examine discriminant validity using HTMT ratios and compare alternative structural models, including higher-order and bifactor CFA models, to better evaluate the distinctiveness and structural organization of the ASB construct.

Furthermore, the results of the convergent validity analysis indicated that scores on the (ASB) scale were positively correlated with levels of depression, anxiety, and stress, supporting the scale’s convergent validity. These findings are consistent with prior research that has identified a significant positive relationship between anti-social behaviors and psychological factors such as depression, anxiety, and stress [3, 6, 12, 19]. This finding can be explained by the fact that individuals with symptoms of depression and anxiety often struggle with emotion regulation and managing social conflicts. Difficulties in impulse control, anger management, and problem-solving through adaptive strategies may lead to aggressive behaviors, irritability, or even withdrawal from social norms, all of which are manifestations of antisocial behavior [2, 19]. Moreover, repeated experiences of stress and psychological pressure may reduce an individual’s emotional resilience, resulting in the emergence of maladaptive and antisocial behaviors as an ineffective coping mechanism to release tension and regain a sense of control. In other words, antisocial behaviors may serve as a temporary “solution” for stress relief, although they tend to exacerbate problems in the long run [18].

According to the results of this study, the ASB questionnaire demonstrated satisfactory internal consistency, test-retest reliability, construct reliability, and convergent validity. The Cronbach’s alpha coefficient for the scale was 0.97, and the two-week test-retest correlation coefficient was 0.72. These findings are consistent with the results of the original scale development study [10]. Furthermore, the CR values above 0.90 and AVE values well above the recommended threshold of 0.50 provide additional evidence that the latent constructs are measured consistently and capture a substantial proportion of the variance in their respective indicators. Although Pearson correlation coefficients were used to estimate test-retest reliability in the present study, future validation studies are encouraged to report intraclass correlation coefficients (ICC), which are generally considered a more comprehensive indicator of temporal stability. Additionally, the item-total correlation coefficients were notably high, with most items demonstrating correlations above 0.90. While this finding indicates that individual items are strongly associated with the overall construct of antisocial behavior and contribute substantially to the measurement of the scale, it may also suggest a degree of overlap or redundancy among some items. Therefore, future studies may consider developing a shorter version of the ASB questionnaire by identifying the most informative items through item response theory (IRT) or other item-reduction approaches while maintaining adequate psychometric properties.

Given that anti-social behavior in a higher education setting may reflect underlying psychological distress, diminished well-being, and functional impairment, future research could benefit from employing ASB assessment tools to identify at-risk students and guide intervention strategies more effectively. In addition to its psychometric utility, the ASB questionnaire may have important practical implications for Afghan universities. Although the present findings provide preliminary support for the applicability of the ASB questionnaire in Afghan higher education settings, its broader practical and clinical utility should be interpreted cautiously. Additional evidence regarding predictive validity, longitudinal stability, responsiveness to intervention, and cross-cultural replication is still needed before widespread implementation in university counseling and mental health services can be recommended. Nevertheless, the ASB questionnaire may offer a potentially useful screening tool for identifying students who could benefit from further psychological assessment or supportive interventions within university settings. Owing to its relative simplicity and ease of administration, the ASB questionnaire may serve as a practical tool for research and preliminary screening. At the same time, the interpretation and application of antisocial behavior measures should remain sensitive to the broader sociocultural context in which such behaviors are expressed.

Beyond the psychometric findings, the interpretation of antisocial behavior in the Afghan university context should also consider the broader cultural, social, and educational environment in which these behaviors occur. In Afghanistan, collectivist social values, strong emphasis on social reputation, and culturally defined expectations of student behavior may influence how antisocial behaviors are expressed and reported. For instance, behaviors such as indirect disruption, passive resistance, or subtle forms of academic misconduct may be shaped by social norms and institutional constraints that differ from those in Western contexts where the ASB scale was originally developed. Additionally, ongoing social and economic challenges may further influence students’ emotional regulation and interpersonal functioning, potentially intensifying certain forms of antisocial behavior. Therefore, while the present findings support the validity of the ASB in this sample, they should be interpreted within the specific cultural and educational context of Afghan universities. This highlights the importance of culturally informed interpretation in cross-cultural scale adaptation studies.

Despite its contributions, the present study is not without limitations. The sample was limited to students from Herat University, which substantially restricts the generalizability of the findings to broader Afghan student populations and other higher education settings. In addition, the present study did not examine measurement invariance across gender. Therefore, it remains unclear whether the ASB questionnaire functions equivalently for male and female students. Future studies should investigate configural, metric, and scalar invariance across gender and other demographic groups to establish the cross-group comparability of the scale. Moreover, the use of convenience sampling may have introduced selection bias and limited the sample’s representativeness. Therefore, the findings should be interpreted with caution and may not generalize to all Afghan university students, particularly those from diverse cultural, socioeconomic, and educational backgrounds. Recruiting participants from multiple universities across different regions of Afghanistan using probability-based sampling strategies would improve the generalizability and representativeness of future findings. Universities across Afghanistan may differ considerably in terms of cultural norms, socioeconomic conditions, educational environments, gender dynamics, and student experiences. Therefore, the psychometric properties and contextual interpretation of the ASB questionnaire observed in this study may not fully represent students from other regions or institutions within the country. In addition, because the sample was recruited from a single university using convenience sampling, the observed factor structure and reliability estimates may partly reflect institution-specific characteristics. Differences in university culture, institutional policies, and campus climate may influence the expression of antisocial behavior and, consequently, the psychometric performance of the ASB questionnaire. Therefore, replication across multiple universities is necessary to confirm the stability and generalizability of the present findings.

Moreover, although the DASS-21 provided a theoretically relevant indicator of emotional distress for examining convergent validity, future studies should employ additional behavioral and personality-based measures more directly related to antisocial behavior to strengthen the construct validation of the ASB further. In particular, future studies may benefit from including theoretically related constructs such as aggression, impulsivity, empathy, psychopathy, moral disengagement, and dark personality traits to provide more comprehensive evidence for convergent and discriminant validity. Additionally, criterion-related validity and discriminant validity were not directly examined in the present study and should be evaluated in future research. In addition, although the translation procedure aimed to ensure conceptual and linguistic equivalence, cultural differences may influence how certain antisocial behaviors, particularly indirect distractive behaviors, are perceived and expressed within Afghan university settings. Therefore, future research should further investigate the cultural appropriateness and contextual interpretation of specific ASB items across different Afghan student populations.

Given the above limitations, it is recommended that, alongside the ASB questionnaire, complementary tools such as the Dirty Dozen Scale (DDS), the short form of the Self-Compassion Scale (SCS), and other related questionnaires be utilized to achieve a more comprehensive and accurate assessment. Also, it is suggested that future studies examine the relationship between ASB and academic underachievement, depression, and social anxiety. Furthermore, it is suggested that subsequent studies enhance the generalizability of the findings by employing larger, more diverse samples, adopting random sampling techniques, and incorporating qualitative methods, such as interviews, to complement and strengthen the data obtained through self-report questionnaires. Future studies may also benefit from incorporating qualitative approaches, such as interviews or cognitive feedback sessions with students, to explore item comprehension, cultural relevance, and subjective interpretations of antisocial behavior in Afghan higher education contexts.

## Conclusion

In conclusion, the findings of this study suggest that the Dari/Farsi version of the Anti-Social Behavior in a higher education setting (ASB) questionnaire demonstrates acceptable psychometric properties among Afghan university students. The scale demonstrated satisfactory internal consistency, temporal stability, construct reliability, convergent validity, and construct validity, suggesting that the Dari/Farsi ASB questionnaire is a psychometrically sound instrument for use among Afghan university students. Nevertheless, the findings should be interpreted with caution due to the use of a convenience sample drawn from a single university and the limited availability of external validation measures within the Afghan context. Despite these limitations, the ASB questionnaire may provide valuable support for researchers, counselors, and student support services in identifying behavioral difficulties and informing preventive and intervention-based strategies in academic settings. For example, university counseling centers may use the ASB as a screening tool to identify students experiencing interpersonal, behavioral, or adjustment difficulties at earlier stages. Mental health professionals and student affairs services may also benefit from incorporating the ASB into psychoeducational programs, behavioral risk assessments, and targeted support interventions aimed at improving students’ emotional regulation, social functioning, and academic adaptation. Future research is encouraged to employ the ASB in longitudinal and intervention-oriented studies to better understand the development, persistence, and modification of antisocial behaviors over time. Furthermore, cross-cultural replication studies in different educational and cultural contexts are recommended to further examine the generalizability, cultural sensitivity, and structural stability of the ASB beyond the Afghan context.

## Data Availability

Data are available upon reasonable request.
